# 4D Printing Self-Morphing Structures

**DOI:** 10.3390/ma12081353

**Published:** 2019-04-25

**Authors:** Mahdi Bodaghi, Reza Noroozi, Ali Zolfagharian, Mohamad Fotouhi, Saeed Norouzi

**Affiliations:** 1 Department of Engineering, School of Science and Technology, Nottingham Trent University, Nottingham NG11 8NS, UK; 2 School of Mechanical Engineering, Faculty of Engineering, University of Tehran, Tehran, Iran; reza.noroozi@ut.ac.ir (R.N.); saeednorouzi@ut.ac.ir (S.N.); 3 School of Engineering, Deakin University, Geelong, Victoria 3216, Australia; a.zolfagharian@deakin.edu.au; 4 Department of Design and Mathematics, the University of the West of England, Bristol BS16 1QY, UK; mohammad.fotouhi@uwe.ac.uk

**Keywords:** 4D printing, shape memory polymer, self-morphing, experiments, FEM

## Abstract

The main objective of this paper is to introduce complex structures with self-bending/morphing/rolling features fabricated by 4D printing technology, and replicate their thermo-mechanical behaviors using a simple computational tool. Fused deposition modeling (FDM) is implemented to fabricate adaptive composite structures with performance-driven functionality built directly into materials. Structural primitives with self-bending 1D-to-2D features are first developed by functionally graded 4D printing. They are then employed as actuation elements to design complex structures that show 2D-to-3D shape-shifting by self-bending/morphing. The effects of printing speed on the self-bending/morphing characteristics are investigated in detail. Thermo-mechanical behaviors of the 4D-printed structures are simulated by introducing a straightforward method into the commercial finite element (FE) software package of Abaqus that is much simpler than writing a user-defined material subroutine or an in-house FE code. The high accuracy of the proposed method is verified by a comparison study with experiments and numerical results obtained from an in-house FE solution. Finally, the developed digital tool is implemented to engineer several practical self-morphing/rolling structures.

## 1. Introduction

In recent years, three-dimensional (3D) printing has dramatically been developed in various industrial fields to construct structures with complicated 3D shapes based on computer-aided design (CAD) models [1]. The process of creating 3D objects was invented in 1986 by Charles Hull and introduced as additive manufacturing (AM), rapid prototyping (RP), or solid-freeform (SFF) [2]. This convenient technology could construct 3D structures with thermoplastic polymer materials such as acrylonitrile butadiene styrene (ABS) [3,4,5], polylactic acid (PLA) [3,5,6], polyamide (PA) [7], and polycarbonate (PC) [8], that were already being used for biomechanics [9], optical metamaterials [10], smart textiles [11], and other applications. The advantages of this fabrication method are the optimal use of the material, a flexible design, and more precise production of complex parts and components.

As a class of multi-scale structures, so-called “metamaterials” exhibit thermo-mechanical properties that are not found in nature. Their unusual characteristics arise from their structures and geometries rather than the material of which they are composed [12]. For the first time, Lakes [13] reported foam structures with negative Poisson ratios. Recently, 3D printing technology has enabled us to fabricate cellular materials with complex architectures [14]. For example, Wang et al. [15] showed dual-material auxetic metamaterials consisting of two parts, stiff walls and elastic joints, that did not show any instability during deformation. The finite element (FE) and experimental results showed that these metamaterials had distinctly different auxeticities and mechanical properties from traditional single-material auxetic metamaterials. In another case, Garcia and et al. [8] designed an all-dielectric uniaxial anisotropic metamaterial and then fabricated and tested it. It was manufactured from polycarbonate using a fused deposition modeling (FDM) 3D printing. Mirzaali et al. [16] used computational models and advanced multi-material 3D printing techniques to rationally design and additively manufacture multi-material cellular solids for which the elastic modulus and Poisson’s ratio could be independently tailored in different directions. Yang et al. [17] used the classical planar tessellation theory to find regular 2D figures that can be used as configurations for first- and second-order honeycombs, and systematically explored the configuration characteristics of the existing two-dimensional (2D) and 3D auxetic and non-auxetic structures. Then, based on a topology analysis, they designed and classified 3D hierarchical metamaterials according to first-order and second-order configurations, which have tailored different ranges of Poisson’s ratio and Young’s modulus. Bodaghi et al. [18] conducted experimental and numerical studies on the mechanical behaviors of metamaterials made of hyperelastics under both tension and compression in a large strain range. They used the FDM method for 3D printing samples and explored metamaterial behaviors in tension and compression modes, revealing buckling instability characteristics. 

**Figure 1:**
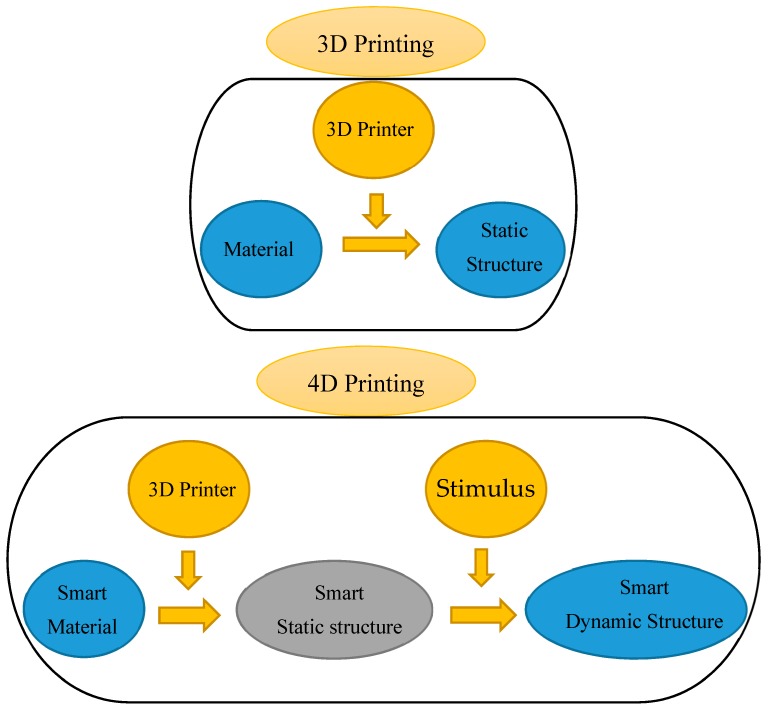
The difference between 3D and 4D printings.

By printing “smart” materials, 3D printing shifts to another level that is called 4D printing. In other words, 4D printing is a combination of 3D printing with time as its fourth dimension [19]. Figure 1 shows the difference between 3D and 4D printings, where in a 4D-printed sample, stimuli like water, heat, a combination of heat and light, and a combination of water and heat trigger actuations. The selection of the stimulus depends on the requirements of the specific application, which also determine the types of smart materials employed in 4D-printed structures. Among active materials, shape memory polymers (SMPs) have more advantages. These advantages are higher recoverable strain of up to 400%, lower density, lower cost, simple procedure for programming of shapes, and good controllability over the recovery temperature. SMPs can hence be utilized in the automotive and aerospace industries, and other fields. For example, Tibbits et al. [20] created a linear strip structure composed of rigid and active materials. This structure could transform into a corrugated structure when it was placed in water. This was a demonstration of 1D-to-2D shape-shifting using the self-bending mechanism. They printed a 2D flat surface with different swelling activated in the water that could change into closed-surface cubes with a 3D shape. Zhang et al. [21] showed that the release of the internal strain in printed samples generated during the 3D printing process made the printed structure remain flat under heating, and when it was cooled to room temperature, it changed into a 3D structure. Jamal et al. [22] showed a configuration change for tissue engineering where a 2D planar bio-origami changed into a 3D pattern by the self-bending operation. This configuration change was enabled by different swelling ratios of hydrogels and rigid materials in the water. By understanding the FDM printing method and SMP cycle, Bodaghi et al. [23] manufactured adaptive metamaterials enabled by functionally graded (FG) 4D printing technology, without application of any programming process and external manipulation. They implemented an in-house FE code to solve constitutive governing equations of SMP structures. Using 4D printing, Joanne et al. [24] studied cross-folding origami structures that were made of multi-material components along different axes and different horizontal hinge thicknesses with a single homogeneous material. Chen et al. [25] demonstrated geometrically reconfigurable, functionally deployable, and mechanically tunable lightweight metamaterials utilizing 4D printing. They introduced metamaterials that were made of photo-crosslinkable and temperature-responsive SMPs. By implementing 4D printing technology, Bodaghi and Liao [26] introduced tunable continuous-stable metamaterials with reversible thermo-mechanical memory operations. Zolfagharian et al. [27] provided a control-oriented modelling approach for 3D-printed polyelectrolyte soft actuators. They developed an electro-chemo-mechanical model for the 3D-printed polyelectrolyte soft actuators and validated it with the experimental data. A new class of metamaterials, so-called “self-morphing structures”, has recently been introduced and studied [28]. Yu et al. [29] designed a new concept of a morphing wing based on SMPs and their reinforced composites. Tao et al. [30] simulated self-folding SMP hinged shells by implementing a complicated user defined material (UMAT) subroutine into the commercial FE software package of Abaqus. 

The literature review implies that researchers have mostly developed their own in-house FE codes or implemented complicated UMATs using FE commercial software packages to model self-folding structures.

This paper aims at introducing self-bending/morphing/rolling structures fabricated by 4D printing technology and simulating their thermo-mechanical behaviors by a novel simple computational tool. The main approach is based on an understanding of thermo-mechanical behaviors of shape memory polymers and the concept behind FDM technology, as well as experiments to explore how printing speed can control self-bending features. The feasibility of the SMP primitives with self-bending features via FG 4D printing is first demonstrated experimentally. The self-folding 1D-to-2D process is simulated by introducing a straightforward method into a commercial FE software package of Abaqus that is much simpler than writing a UMAT subroutine or an in-house FE solution. 4D printing and the computational tool are applied to develop practical complex structures with self-bending/morphing/rolling features. A good qualitative and quantitative correlation is observed, verifying the accuracy of the proposed method.

## 2. Conceptual Design

### 2.1. FG 4D Printing 

**Figure 2:**
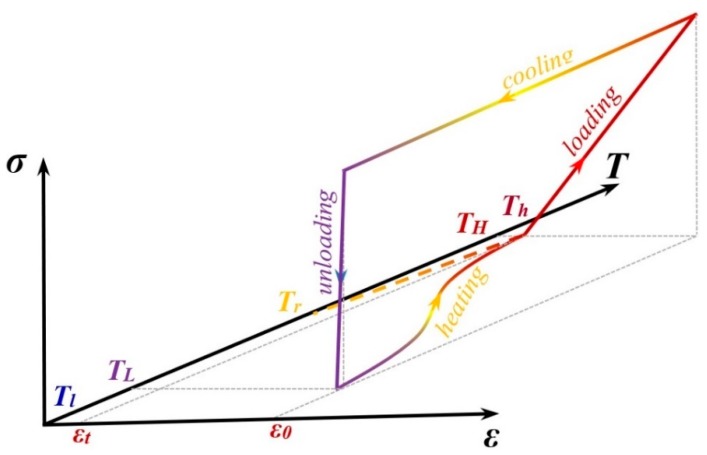
Shape memory polymer (SMP) thermo-mechanical cycle.

In this section, inspired by SMP features, we show the potentials of 3D printing in the design and development of the adaptive metamaterials. SMPs can retain a temporary shape and recover into their original shape when subjected to an environmental stimulus such as heat. Figure 2 shows an SMP thermo-mechanical cycle. The polymer is initially at temperature $T_{r}$. First, the polymer is heated up to temperature $T_{h}$ that is greater than the glassy temperature of the polymer $(T_{h}>T_{g}$). Then, the material undergoes a strain level of ε_0_ due to the applied load (loading). Next, it is fixed in the strain ε_0_ while reducing the temperature to $T_{L}$, which is lower than $T_{g}$ (cooling process). Afterwards, at the constant temperature $\;T_{L}$, the mechanical constraint is released (unloading). Upon heating, the pre-strain releases and the permanent shape is recovered (this is called stress-free strain recovery). 

**Figure 3:**
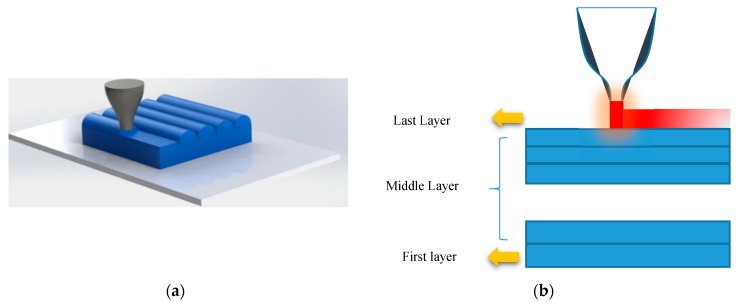
Schematic of the fused deposition modeling (FDM) method.

Among various 3D printing technologies, FDM technology applies a similar thermo-mechanical process to the material during printing. Figure 3 shows a schematic of the FDM method. The material is heated inside the liquefier at a temperature (*T_in_*) that is greater than its *T_g_*. It is then placed onto the platform by the printer head moving at the speed $\;S_{p}$. In fact, in this process, the material is stretched at a high temperature similar to the heating–loading process described for the SMP (heating and loading) producing a pre-strain. After placing each layer on the platform, the printed layer is cooled and solidified. This stage is like the SMP cooling step. Once a layer is printed, the build platform advances downward and the printing head proceeds to place the next layer. Finally, the thermo-mechanical programming process is completed by removing the 3D-printed object from the platform (mechanical unloading).

It is worth noting that, when printing the second layer, the hot material is placed on the first layer and reheats it. By partially reheating the first layer, some of the pre-strain is released. In a similar way, by printing other layers, the bottom layers are always reheated at each stage of printing and some of their pre-strains are released. Therefore, the first layer has the least pre-strain and the last layer has the maximum pre-strain as it never gets any heat, since the nozzle leaves it at the end of the 3D printing step. It can therefore be concluded that the printing speed can be considered as a control parameter that affects pre-strain values in the printed layers. This programming is performed in an FG manner, as the pre-stain can be changed layer-by-layer gradually. 

### 2.2. Materials and Printing

In this study, PLA filaments with diameters of 1.75 mm and glass transition temperatures of 63 °C are used. All the samples are manufactured with 3D printing and the FDM method. The extruder diameter is 0.4 mm and the liquefier temperature is set at 190 °C. The temperatures of the platform and chamber are set at room temperature and 24 °C, respectively. The 3D printing is performed at three different speeds, namely 20, 40, and 70 mm/s. The layer height is set to 0.1 mm.

**Figure 4:**
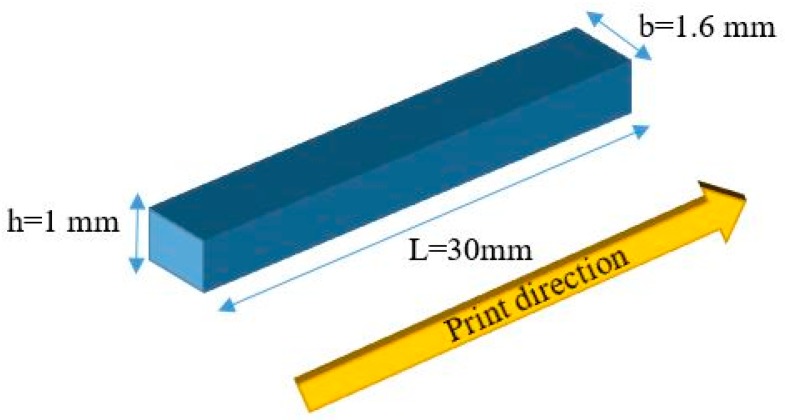
Geometry of the 3D-printed sample.

The thermo-mechanical properties of the PLA are provided in this section. Dynamic mechanical analysis (DMA) (Q800 DMA, TA Instruments, New Castle, DE, USA) is performed to specify temperature-dependent material properties of the PLA. For this purpose, the sample is printed in the dimensions of 30, 1.6, and 1 mm for length, width, and thickness, respectively. Figure 4 shows the geometry of the 3D-printed sample and print direction.

**Figure 5:**
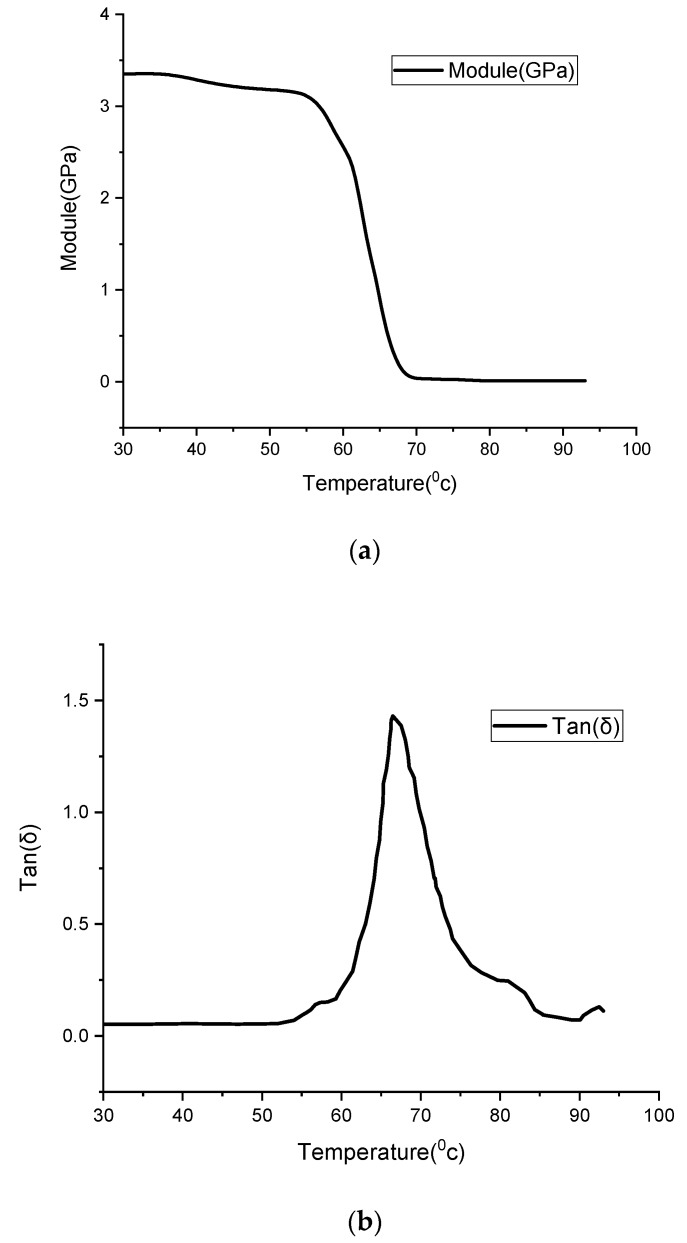
Dynamic mechanical analysis (DMA) results for polylactic acid (PLA): (**a**) Storage modulus $E^{\prime }$, (**b**) tan(δ).

A DMA test is performed in an axial tensile condition with 1 H of force oscillation frequency and a 5 °C/min heating rate, with the temperature ranging from 30 to 93 °C. The results of the DMA test in terms of storage modulus *E´* and tan(δ) are shown in Figure 5a,b, respectively. Also, the numerical results of the DMA test are presented in Table 1.

## 3. Theoretical Modeling 

### 3.1. SMP Model

Shape memory polymers are a new class of materials that can keep a temporary shape and return to their original shape upon application of a stimulus such as temperature. They consist of glassy and rubbery phases. Thus, we can show the volume fraction of the rubbery and glassy phases by scalar variables $\xi_{g}$ and $\xi_{r}$ as:

(1) $$\xi_{g}=\frac{V_{g}}{V}\;\;\;\xi_{r}=\frac{V_{r}}{V}\;$$

where $V_{g}$ is the glassy phase volume and $V_{r}$ denotes the rubbery phase volume. The summation of the volume fractions of the two phases should equal to unity ($\xi_{g}+\xi_{r}=1$). The transformation of the rubbery phase into the glassy phase is considered to be only a function of temperature. Thus, $\xi_{g}$ and $\xi_{r}$ only depend on the temperature. The volume fraction of the rubbery phase can be written in terms of the glassy volume fraction as:

(2) $$\xi_{r}=1-\xi_{g}$$

We also assume that $\xi_{g}$ is an independent variable and can be expressed as:

(3) $$\xi_{g}=\xi_{g}(T)\;\;\;V_{g}=V_{g}(T)\;$$

Considering the experimental DMA test results, the glassy volume fraction can be interpolated by an explicit function as:

(4) $$\xi_{g}=\frac{\tanh(a_{1}T_{g}-a_{2}T)-\tanh(a_{1}T_{g}-a_{2}T_{h})}{\tanh(a_{1}T_{g}-a_{2}T_{h})-\tanh(a_{1}T_{g}-a_{2}T_{1})}$$

where $a_{1},a_{2}$ are chosen to fit the DMA curve.

The rubbery and glassy phases in SMPs are assumed to be linked in series. Considering a small strain regime, justified by the fact that the printed structures experience small strains and moderately large rotations, additive strain decomposition is adopted as:

(5) $$\varepsilon =\xi_{g}\varepsilon_{g}+(1-\xi_{g})\varepsilon_{r}+\varepsilon_{in}+\varepsilon_{th}$$

where ε denotes total strain; $\varepsilon_{g}$ and $\varepsilon_{r}$ indicate strain of the glassy and rubbery phases, respectively; $\varepsilon_{in}$ is the inelastic strain due to phase transformation; and $\varepsilon_{th}$ denotes the thermal strain which is defined as:

(6) $$\varepsilon_{th}=\int_{T_{0}}^{T}\alpha_{e}(T)\;dT$$

where $T_{0}$ is the reference temperature and $\alpha_{e}$ is the equivalent thermal expansion, defined as:

(7) $$\alpha_{e}=\alpha_{r}+(\alpha_{g}-\alpha_{r})\;\xi_{g}(T)$$

During the cooling process, the rubbery phase transforms into the glassy phase and its strain, $\varepsilon_{in}$, is stored in the material. It is formulated as:

(8) $${\dot{\varepsilon }}_{in}={\dot{\xi }}_{g}\;\varepsilon_{r}$$

in which the dot denotes the rating function.

In the heating process, the stored strain is given to be released gradually in proportion to the volume fraction of the glassy phase with respect to the preceding glassy phase. The strain storage is expressed as:

(9) $${\dot{\varepsilon }}_{in}=\frac{{\dot{\xi }}_{g}}{\xi_{g}}\;\varepsilon_{in}$$

To derive the stress state, the second law of thermo-dynamics in the sense of the Clausius–Duhem inequality should be satisfied. In this model, ε and T are selected as external control variables, while $\varepsilon_{g},\varepsilon_{r},\varepsilon_{in}$, and $\xi_{g}$ are internal variables. Considering Helmholtz free energy density functions, stress σ can be derived as:

(10) $$\sigma =\sigma_{g}=\sigma_{r}$$

where

(11) $$\sigma_{g}=C_{g}\varepsilon_{g}\;,\;\sigma_{r}=C_{r}\varepsilon_{r}$$

where *C* is the elasticity matrix defined as:

(12) $$C=\frac{E}{\left(1+\nu )(1-2\nu \right)}\;\left[\begin{array}{cccccc} 1-\nu & \nu & \nu & 0 & 0 & 0\\ \nu & 1-\nu & \nu & 0 & 0 & 0\\ \nu & \nu & 1-\nu & 0 & 0 & 0\\ 0 & 0 & 0 & \frac{\left(1-2\nu \right)}{2} & 0 & 0\\ 0 & 0 & 0 & 0 & \frac{\left(1-2\nu \right)}{2} & 0\\ 0 & 0 & 0 & 0 & 0 & \frac{\left(1-2\nu \right)}{2} \end{array}\right]$$

By substituting Equation (11) in Equation (5), we obtain the stress as:

(13) $$\sigma =C_{e}(\varepsilon -\varepsilon_{in}-\varepsilon_{th})$$

where $C_{e}$ is the equivalent elasticity tensor and is expressed as:

(14) $$C_{e}={\left(S_{r}+\xi_{g}(S_{g}-S_{r})\right)}^{-1}$$

in which S denotes the inverse matrix of C($S=C^{-1}$), so-called the “compliance matrix”. 

The non-linear SMP behavior can be treated as an explicit time-discrete stress/strain-temperature driven problem. The time domain [0,t] is divided into subdomains, and the equation is solved in the local domain $\left[t^{k},t^{k+1}\right]$. The superscript k+1 for all variables denotes the current step, while the superscript *k* indicates the previous step. The inelastic strain can be calculated by applying the linearized implicit backward Euler integration method to the flow rule. Thus, Equations (8) and (9) can be discretized as:

(15) $$\varepsilon_{in}^{k+1}=\varepsilon_{in}^{k}+\Delta \xi_{g}^{k+1}\;\varepsilon_{r}^{k+1}$$

(16) $$\varepsilon_{in}^{k+1}=\varepsilon_{in}^{k}+\frac{\Delta \xi_{g}^{k+1}}{\xi_{g}^{k+1}}\;\varepsilon_{in}^{k+1}$$

where

(17) $$\Delta \xi_{g}^{k+1}=\xi_{g}^{k+1}-\xi_{g}^{k}$$

By substituting Equations (11) and (13) into Equations (15) and (16) along with a mathematical simplification, we can explicitly update the inelastic strain for the cooling and heating processes. For the cooling process, we can write: 

for stress control:

(18) $$\varepsilon_{in}^{k+1}=\varepsilon_{in}^{k}+\Delta \xi_{g}^{k+1}S_{r}^{k+1}\sigma^{k+1}$$

for strain control:

(19) $$\varepsilon_{in}^{k+1}={\left(I+\Delta \xi_{g}^{k+1}S_{r}^{k+1}C_{e}^{k+1}\right)}^{-1}\;(\varepsilon_{in}^{k}+\Delta \xi_{g}^{k+1}\;S_{r}^{k+1}C_{e}^{k+1}\;(\varepsilon^{k+1}-\varepsilon_{th}^{k+1}))\;$$

For the heating process, Equation (16) can be simplified as:

(20) $$\varepsilon_{in}^{k+1}=\frac{\xi_{g}^{k+1}}{\xi_{g}^{k}}\varepsilon_{in}^{k}$$

Now, by substituting the updated inelastic strain into Equation (15), the stress–strain relationship for heating and cooling processes can be obtained as:

(21) $$\sigma^{K+1}=C_{D}^{k+1}(\varepsilon^{k+1}-\delta \varepsilon_{in}^{k+1}-\varepsilon_{th}^{k+1})$$

where elasticity tensor $C_{D}$ and the δ parameter for the heating and cooling processes are defined as:

(22) $$\begin{array}{l} C_{D}^{k+1}={\left(I+\Delta \xi_{g}^{k+1}S_{r}^{k+1}C_{e}^{k+1}\right)}^{-1}\;C_{e}^{k+1},\;\delta =1\;\dot{T}<0\\ C_{D}^{k+1}=C_{e},\;\delta =\frac{\xi_{g}^{k+1}}{\xi_{g}^{k}}\;\;\;\;\;\;\;\;\;\;\dot{T}>0\; \end{array}$$

### 3.2. FE Methodology 

#### 3.2.1. In-house FE method

A Ritz-based FE solution is implemented to predict the thermo-mechanical behaviors of FG 4D-printed structures. A 3D twenty-node quadratic serendipity hexahedron element is applied to this problem. It has twenty nodes so that eight corner nodes are augmented with twelve side nodes located at the side center. The element also has three degrees of freedom per node ($u_{i}$(i=1,2,3). For more details on the FE modeling, one may refer to [23].

#### 3.2.2. FE Abaqus

The results of the DMA test in the form of a temperature-dependent modulus are introduced in the FE Abaqus software. Table 2 shows the value of Young’s modulus at different temperatures.

**Figure 6:**
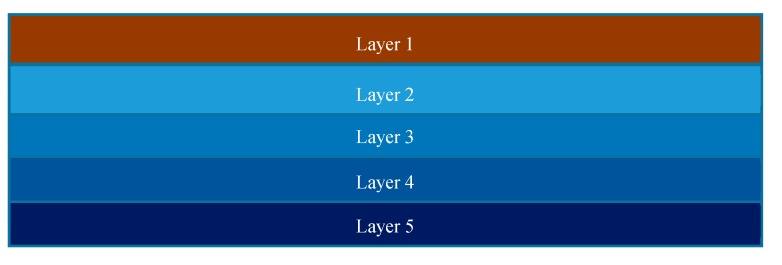
Discretized straight beam-like sample.

To model the straight beam-like samples with FG features, they are discretized to five sections where each section has a different thermal expansion coefficient. Figure 6 shows a straight beam-like sample in a discretized form with different thermal expansion coefficients.

In Figure 6, layer 1 indicates the last printed layer, while layer 5 is the first layer that has been 3D-printed.

## 4. Results and Discussion

**Figure 7:**
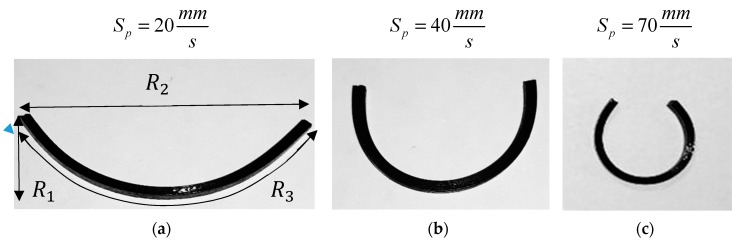
Deformed configurations of the beams printed with different speeds after the heating–cooling process.

In this section, the experimental and numerical analyses of the self-bending/morphing structures are presented. The samples are heated by dipping into the hot water at a prescribed temperature of 85 °C that is greater than the transition temperature by 22 °C. Three straight beam-like samples with dimensions of (30 × 1.6 × 1) mm are printed at liquefier temperature *T_in_* = 190 °C and the print speed of $S_{p}=20\;mm/s$, $S_{p}=40\;mm/s$ and $S_{p}=70\;mm/s$, respectively. The configuration of the three samples after the heating–cooling process is depicted in Figure 7. As it can be seen, the samples self-bend when dipping in the hot water. The observed self-bending is due to an unbalanced pre-strain induced during 3D printing and deposited through the thickness direction. Unbalancing in the through-the-thickness pre-strain distribution leads to a mismatch in the free-strain recovery, producing curvatures and revealing a transformation from a temporary straight shape to a permanent curved shape. It should be mentioned that curved beams could be manually programmed again to get another temporary shape and reveal shape memory effects upon heating. It is also found that the pre-strain has an increasing trend through the thickness from the lower to the upper layer that leads the beam to be changed upward. This self-bending is such that the samples with higher printing speeds have larger bending angles. One of the reasons that can explain this trend is that more speed provides more mechanical loading that may induce more pre-strain. In fact, the FDM printing process shows the capability of both fabrication and hot programming at the same time. To characterize the deformed shape, we define three geometric parameters $\left(R_{1},R_{2},R_{3}\right)$ that can describe the deformed shapes. $R_{3}$, $R_{2}$ and $R_{1}$ denote the outer length, the opening, and the depth of the mid-surface of the deformed sample, respectively. 

**Figure 8:**
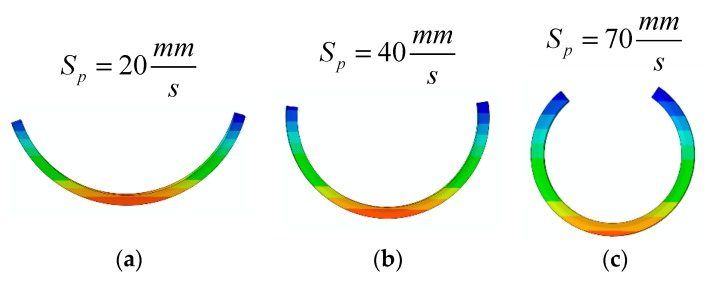
The FE Abaqus simulation of the self-bending beams illustrated in Figure 7.

Then, the FE Abaqus software is used to model the printed samples. For this purpose, the element type C3D8T is used, and the sample is discretized into five sections with different thermal expansion coefficients. The thermal expansion coefficient on each layer is chosen to obtain the deformed configuration for the specific printing speed. Table 3 shows the thermal expansion coefficient of each layer for different printing speeds. Figure 8 also shows the results from the FE Abaqus simulation.

Table 4 also lists the geometric parameters obtained from the experiments, FE Abaqus, and in-house FE code. It is found that simulation results of Abaqus are in a good agreement with the characteristics observed in the experiments and the in-house FE solution. It validates the reliability of the SMP programming by considering FG thermal expansion in the FE Abaqus.

**Figure 9:**
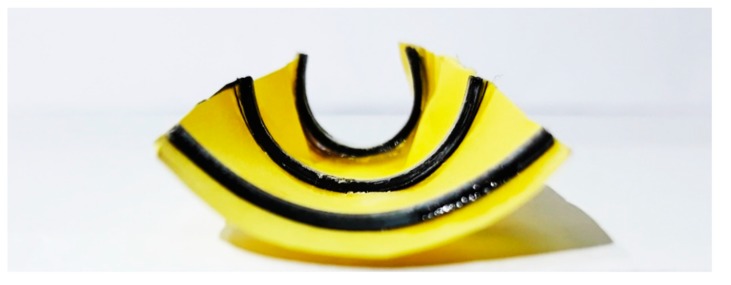
Configuration of the flat sheet reinforced by three straight 4D-printed beams after the heating–cooling process.

Next, potential applications of self-bending primitives are demonstrated. First, we design a flat sheet with dimensions of (50 × 30) mm, reinforced by three straight beams that are printed with different speeds. Figure 9 shows the experimental results of the deformed configuration of the structure after heating up to 65 °C and then cooling down to room temperature. Young’s modulus of the PLA at 85 °C is very low. Therefore, if the structure is heated up to 85 °C, the beams become very soft and the paper sheet under tension returns the beams to the undeformed configuration. That is why the structure should be heated up to 65 °C. As observed in Figure 9, the bending angles are different for the beams printed with different speeds. Due to this fact, the sheet is bent along the central line with different angles and deformed into a conical panel by heating. This can be considered as a demonstration of a 2D-to-3D shape-shifting by the self-morphing mechanism.

**Figure 10:**
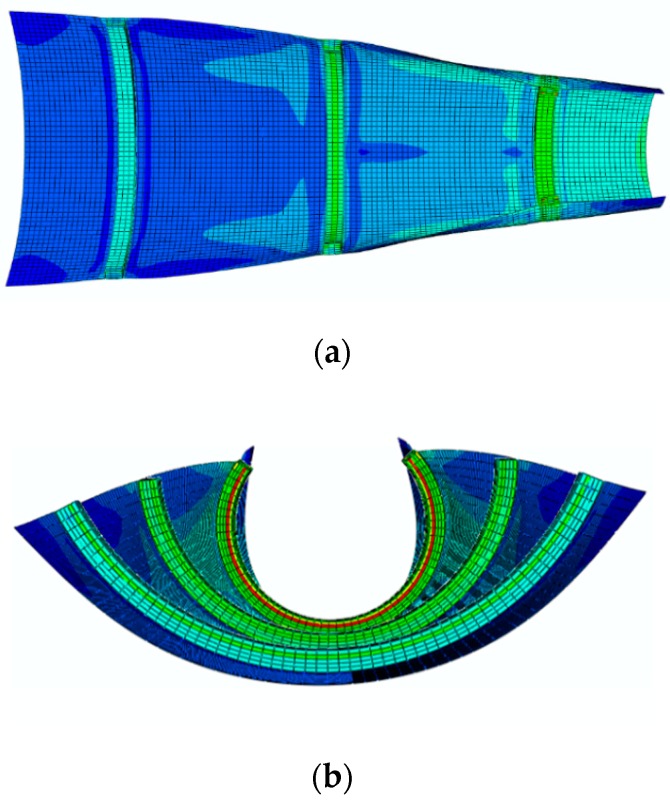
The FE Abaqus simulation of the self-morphing composite structure illustrated in Figure 9.

The composite structure, including the main flat sheet reinforced by three straight beams with different pre-strain levels, is modeled by the FE Abaqus. The interaction between the beams and the paper sheet is of the Tie type, which is a perfect bond between the beams and the paper sheet. After determining the thermal boundary conditions similar to the experimental conditions, the structure is heated up to 65 °C and then cooled down to the room temperature. Figure 10 represents simulation results of the deformed configuration that properly match with the experimental shape. As expected, the beam that is 4D printed with a lower speed has a lower bending angle, while the beams printed faster produce greater bending angles. Similar to the experimental results, the paper sheet bends and transforms into a conical panel with the self-morphing feature.

**Figure 11:**
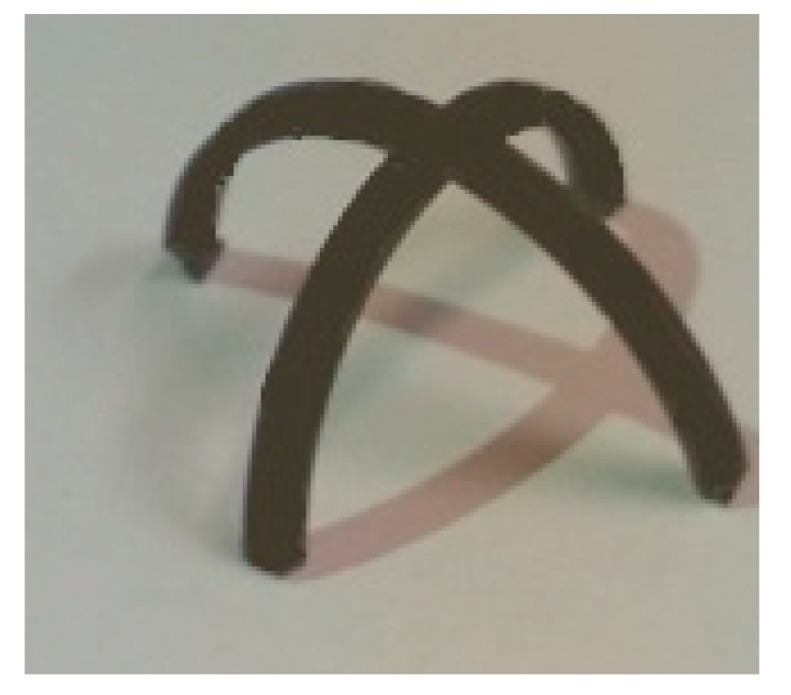
Deformed configuration of the flat plus-like structure after the heating–cooling process.

**Figure 12:**
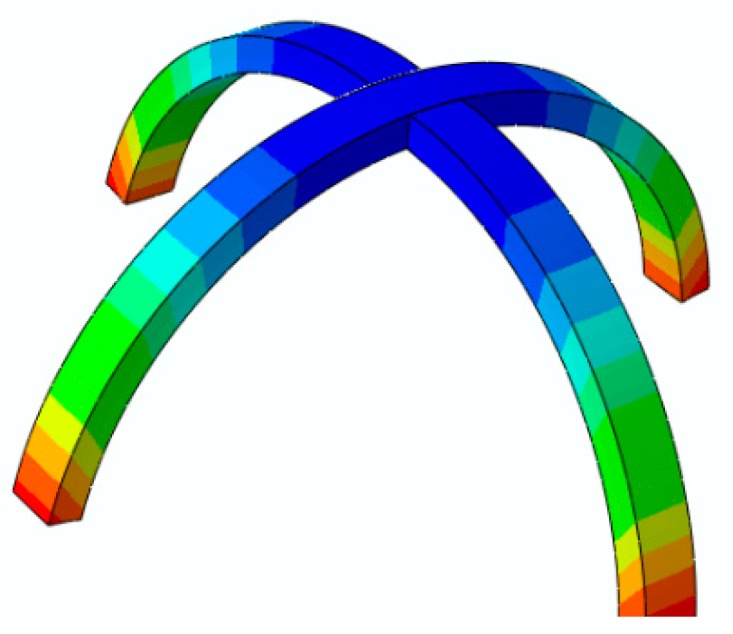
The FE Abaqus simulation of the self-bending gripper illustrated in Figure 11.

For the second example, we consider a plus-like structure printed at $S_{p}=40\;mm/s$. This structure consists of two perpendicular beams with dimensions of (30 × 1.6 × 1) mm. The structure is heated up to 85 °C and then cooled down to room temperature. Figure 11 illustrates the experimental results of the deformed configuration after the heating–cooling process. It can be found that this element has the potential to be used as a flexible self-bending gripper for future mechanical/biomedical devices fabricated by the 4D printing technology. The bending of the gripper can be controlled by changing the printing speed. For example, if a sample is printed at $S_{p}=70\;mm/s$, the bending of the gripper becomes greater. This means that the printing speed can be manipulated to get a desired angle. To model this structure with the FE Abaqus, it is divided into five sections through its thickness, and the thermal boundary conditions are chosen similar to the experimental conditions. The plus-like structure is heated up to 85 °C and then cooled down to room temperature. Figure 12 shows the deformed configuration obtained from the simulation. 

The comparison studies in Figure 7, Figure 8, Figure 9, Figure 10, Figure 11 and Figure 12 revealed the high accuracy of the 3D FE method in Abaqus in replicating the experimental observations. In the following studies, this digital Abaqus tool is implemented to simulate various self-bending devices.

**Figure 13:**
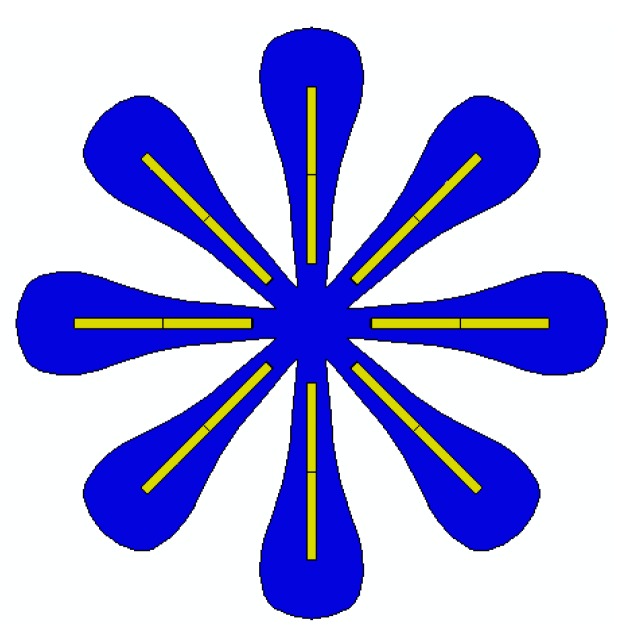
Undeformed configuration of a flower-shaped structure.

**Figure 14:**
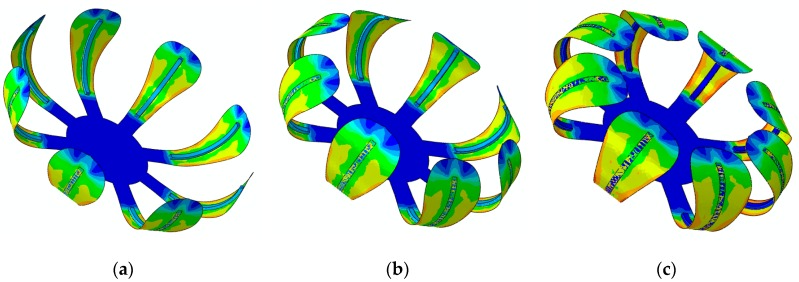
FE Abaqus simulation of the self-morphing flower-shaped structure after the heating–cooling process: (**a**) $S_{p}=40\;mm/s$; (**b**) $S_{p}=70\;mm/s$; (**c**) $S_{p}=70\;mm/s$ (the edge shown on the outer layer of the paper sheet demonstrates the sample place on the interior layer).

Figure 13 shows a flower-like structure composed of a flat paper sheet and eight straight beams. The dimensions of the beams are (30 × 1.6 × 1) mm. The printed beams are at a 10 mm distance from the center of the structure. The beam structures are printed on the paper such that the first printed layer is directly connected to the paper. To model the interactions between the beams and the paper sheet, a Tie-type interaction is assumed. The beam-like structures are 4D printed with different speeds for three different case studies. The configuration of the flower-shaped structure reinforced with beams printed with different speeds after the heating–cooling process is displayed in Figure 14. As it can be seen, when the structure is heated up to 65 °C, it is bent towards the interior layer and the structure transforms into a flower shape.

**Figure 15:**
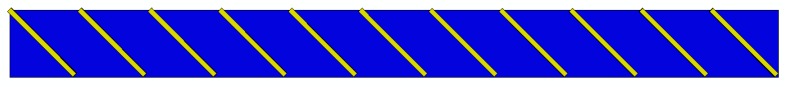
Configuration of the rectangular paper sheet with patterned oblique beams.

**Figure 16:**
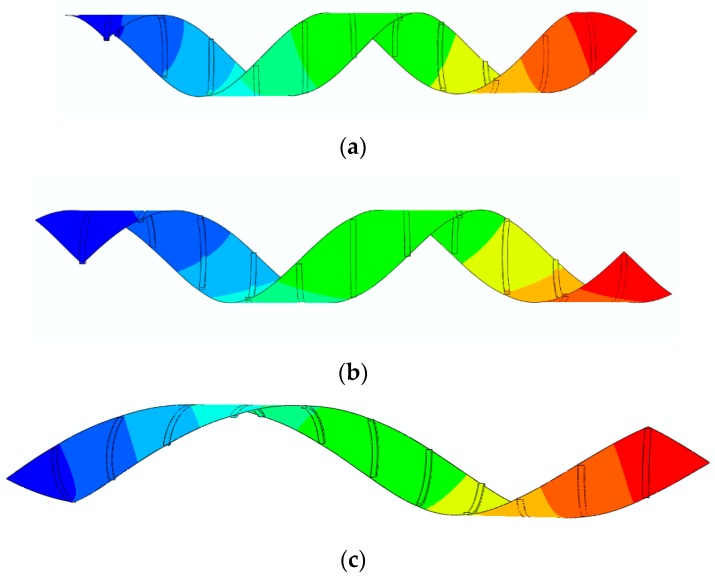
The FE Abaqus simulation of the self-rolling helix after the heating–cooling process: (**a**) $S_{p}=20\;mm/s$, (**b**) $S_{p}=40\;mm/s$, (**c**) $S_{p}=70\;mm/s$ (the edge shown on the outer layer of the paper sheet demonstrates the sample place on the interior layer).

The results presented in Figure 14a show that the deformed configuration for the case of $S_{p}=20\;mm/s$ has a lower bending angle. As it can be observed, by increasing the printing speed, the flower further closes. As another example, a bunch of beams with dimensions of (30 × 1.6 × 1) mm are diagonally printed over a rectangular paper sheet with dimensions of (230 × 21) mm, as shown in Figure 15. The angle between the paper sheet and beams is 45°. The beams are connected to the paper such that the first printed layer is directly connected to the paper. The interaction between the paper sheet and beams is of the Tie type. This structure is heated up to 65 °C and then cooled down to the room temperature. The beam-like structures are 4D printed with different speeds for three different case studies. Figure 16 illustrates the configuration of the rectangular paper sheet reinforced with the beams fabricated with different speeds after the heating–cooling process.

Figure 16 reveals that the structure, initially in a flat state, transforms into a helix upon heating, revealing a self-rolling feature. It is observed that enhancing the printing speed increases the pitch. Therefore, by changing the printing speed, the pitch can be controlled. Moreover, by changing the angle between the paper sheet and the 4D-printed beams, the geometry of the self-rolling helix could be changed.

## 5. Conclusions

The aim of this paper was to develop self-bending/morphing/rolling structures fabricated by FG 4D printing and introduce a novel simple computational tool for replicating their thermo-mechanical behaviors. The concept was based on the understanding of SMP thermo-mechanics and programming the material via common FDM 3D printing technology. Structural primitives with self-bending 1D-to-2D features were 4D printed and then employed as actuation elements to engineer complex structures with 2D-to-3D shape-shifting via self-bending/morphing/rolling mechanisms. The influences of the printing speed on the self-morphing characteristics were investigated in detail. 1D-to-2D and 2D-to-3D shape transformations were simulated by introducing a straightforward method into the commercial FE software package of Abaqus that is much simpler than writing a UMAT subroutine or an in-house FE solution. The 4D-printed materials were modeled as FG materials whose thermal expansions varied through the thickness direction. The accuracy of the proposed approach was verified by a comparison study with experiments and results obtained from the in-house FE solution. Due to the absence of a similar concept, numerical approach, or results in the specialized literature, this paper is likely to pave the way for designing self-bending/morphing/rolling adaptive structures by 4D FDM printing technology.

## Figures and Tables

**Table 1:** DMA results for PLA.

***T* (°C)**	**30**	**40**	**50**	**52**	**54**	**56**	**60**	**62**	**64**	**66**	**68**
Storage module	0.0524	0.055	0.0540	0.0561	0.0701	0.1134	0.2116	0.383	0.6753	1.2890	1.2295
tan(δ)	3.3510	3.288	3.1880	3.1682	3.1402	3.0777	2.6378	2.0433	1.2807	0.5393	0.1459
***T*** **(°C)**	**70**	**72**	**74**	**76**	**78**	**80**	**82**	**84**	**88**	**90**	**93**
Storage module	0.9875	0.658	0.434	0.3292	0.2758	0.2483	0.221	0.1398	0.0774	0.0723	0.1248
tan(δ)	0.030	0.026	0.0218	0.0138	0.0117	0.0117	0.0117	0.0117	0.0117	0.0117	0.030

The peak in the graph of tan(δ) shows the glass transition temperature that is read as $T_{g}=66$ °C.

**Table 2:** Young’s modulus for different temperatures implemented in the finite element (FE) Abaqus.

T (°C)	30	40	50	60	70	80	90
*E* (*MPa*)	3350	3280	3166	2554	48	18	14

**Table 3:** Thermal expansion coefficients for different printing speeds.

*α_i_*(1/°C)	*S_p_* *(mm/s)*		
	20	40	70
*α*_1_	−0.00400	−0.00480	−0.00680
*α*_2_	−0.00320	−0.00384	−0.00544
*α*_3_	−0.00240	−0.00288	−0.00408
*α*_4_	−0.00160	−0.00192	−0.00272
*α*_5_	−0.00080	−0.00096	−0.00136

**Table 4:** Geometric parameters of the beams printed with different speeds after the heating–cooling process.

Method	*V_p_* (mm/s)	*R*_1_ (mm)	*R*_2_ (mm)	*R*_3_ (mm)
Experiments	20	8.20	21.00	29.50
	40	9.60	12.40	29.10
	70	10.70	5.90	28.50
FE Abaqus	20	8.31	21.20	29.55
	40	9.75	12.34	29.00
	70	10.57	6.08	28.60
In-house FE method	20	8.32	21.30	29.47
	40	9.79	12.41	29.01
	70	10.66	6.02	28.49
